# Seronegative autoimmune autonomic ganglionopathy from dual immune checkpoint inhibition in a patient with metastatic melanoma

**DOI:** 10.1186/s40425-019-0748-0

**Published:** 2019-10-17

**Authors:** Catherine A. Gao, Urs M. Weber, Aldo J. Peixoto, Sarah A. Weiss

**Affiliations:** 1 Department of Medicine, Yale University School of Medicine, 333 Cedar Street, New Haven, CT 06520 USA; 2 Department of Medicine (Nephrology), Yale University School of Medicine, 330 Cedar Street, Boardman 114, New Haven, CT 06520 USA; 3 Department of Medicine (Medical Oncology), Yale University School of Medicine, 333 Cedar Street, New Haven, CT 06520 USA

**Keywords:** Immune checkpoint inhibitors, Ipilimumab, Nivolumab, Autoimmune, Autonomic ganglionopathy, Melanoma

## Abstract

**Background:**

Immune checkpoint inhibitors have improved clinical outcomes including survival in several malignancies but have also been associated with a range of immune-related adverse events (irAEs). Neurological irAEs are rare compared to the more typical skin, gastrointestinal, and endocrine toxicities, and are often underrecognized and challenging to diagnose. Here, we report a case of seronegative autoimmune autonomic ganglionopathy (AAG) induced by dual immune checkpoint inhibitor therapy (ICI) in a patient with metastatic melanoma.

**Case presentation:**

A patient with metastatic melanoma was treated with ipilimumab and nivolumab. He developed a constellation of new symptoms including nausea, fatigue, and severe orthostatic hypotension refractory to fluid resuscitation. An infectious, cardiac, neurologic, and endocrine workup were unrevealing. Cardiovascular autonomic testing revealed poor sympathetic nervous system responses. He was diagnosed with seronegative AAG and significantly improved with immunomodulatory therapies including IVIG and steroids as well as varying doses of midodrine and fludrocortisone. He was able to restart nivolumab without recurrence of his symptoms. However, the AAG reoccurred when he was re-challenged with ipilimumab and nivolumab due to disease progression. While the AAG was manageable with steroids at that time, unfortunately his melanoma became resistant to ICI.

**Conclusions:**

Immune checkpoint inhibitors can have a wide range of unusual, rare irAEs, including neurotoxicity such as AAG. Clinicians should maintain suspicion for this toxicity so that treatment can be rapidly provided to avoid disability.

## Background

Monoclonal antibodies against the immune checkpoints cytotoxic T-lymphocyte-associated antigen-4 (CTLA-4) (ipilimumab) and programmed-death-1 (PD-1) (nivolumab, pembrolizumab) have the potential to induce long-term durable responses in patients with advanced melanoma [1–4]. Dual checkpoint inhibition with ipilimumab and nivolumab has led to 3-year overall survival rates of over 50%, but these improved clinical outcomes can be at the expense of immune-related toxicity. The rate of grade > 3 adverse events for patients treated with combination ipilimumab and nivolumab is greater than 50% [5]. While the most common toxicities impact the skin, gastrointestinal tract, and endocrine organs and are well-characterized, rare but serious neurological immune-related adverse events (irAE) have been described [6]. Neurotoxicity attributable to immune checkpoint inhibitors (ICI) is estimated to occur in up to 3% of patients [7, 8] and represents a heterogeneous constellation of syndromes including Guillan-Barre, peripheral neuropathies, myasthenia gravis, and encephalitis among others [9]. Here, we report a case of seronegative autoimmune autonomic ganglionopathy (AAG) induced by dual checkpoint inhibition in a patient with metastatic melanoma. To our knowledge, this is the first case of AAG attributed to ICI reported in the literature.

## Case presentation

A 60-year-old man initially presented with rectal bleeding and discomfort. On physical exam, a rectal mass was initially identified as hemorrhoids. Hemorrhoidectomy was performed, and pathology showed an over 20 mm thick ulcerated mucosal melanoma extending to the margins with a high mitotic rate and the presence of lymphovascular invasion. Tumor profiling showed the malignancy to be BRAF wild-type and KIT mutated (D579 deletion). Upon referral to our institution, staging CT scans showed an enlarging anal mass, a right inguinal mass, and multiple pulmonary nodules consistent with metastatic disease. He underwent palliative trans-anal excision of the rectal mass and was urgently started on dual ICI with ipilimumab 3 mg/kg and nivolumab 1 mg/kg once every 3 weeks for a total of four planned doses. After the third cycle, he presented with a constellation of new symptoms including nausea, constipation, weight loss, fatigue, and hypotension (seated systolic BP as low as 70 mmHg systolic). ICI was held, and he was admitted for further work-up.

His blood pressure did not respond to an initial intravenous fluid challenge of 5 l of normal saline. There were no localizing signs of infection, leukocytosis, tachycardia, or fever, so both sepsis and cytokine release syndrome were felt to be unlikely. His examination was negative other than for orthostatic hypotension. His pupillary responses to light and accommodation, and motor and sensory examinations were normal. A cardiac workup with transthoracic echocardiogram showed preserved ejection fraction without diastolic dysfunction, no significant valvular disease, and no pericardial effusion. A cardiac MRI had no acute findings. An endocrinopathy was considered, however multiple morning cortisol levels were normal as were TSH and a comprehensive evaluation of pituitary function including LH, FSH, prolactin, and GH, thereby ruling out hypopituitarism. There was also no evidence of mineralocorticoid deficiency (normal aldosterone and renin). Other etiologies of autonomic neuropathy were investigated including a work-up for autoimmune (ANA, creatinine kinase), infectious (Lyme, syphilis, HIV), and neurologic (anti-cholinergic receptor antibodies, anti-GAD65 antibody) causes, nutritional deficiencies (B12), and paraneoplastic syndromes (Mayo Clinic paraneoplastic antibody panel), all of which were negative (Table 1). MRI of the brain was negative for intracranial metastases and had no abnormalities that could explain his symptoms. There was no family history of dysautonomia, synucleopathies, or other neurologic disorders.

**Table 1:** Lab test results

Lab	Result	Normal range
Aldosterone	13 ng/dL	3–16 ng/dL
Plasma renin activity	0.51 ng/mL/h	0.25–5.82 ng/mL/h
TSH	3.500 uLU/mL	0.300–4.200 uLU/mL
Free T4	1.12 ng/dL	0.8–1.80 ng/dL
Total T3	69.9 ng/dL (slightly low likely due to impaired T4 to T3 conversion in illness)	79–149 ng/dL
Prolactin	20.1 ng/mL (mildly elevated thought to be from chronic prochlorperazine use)	0–14.0 ng/mL
FSH	7.1 mU/mL	1–12 mU/mL
LH	7.1 mU/mL	1.6–9.6 mU/mL
AM cortisol	multiple unstimulated cortisol levels as high as 22.3	7–25 μg/dL
Testosterone total	334 ng/dL	250–1100 ng/dL
Free testosterone	60.7 pg/mL	35–155 pg/mL
Sex hormone binding globulin	61 nmol/L	22–77 nmol/L
ANA by IFA	< 1:80	< 1:80
B12	1187 pg/mL	180–914 pg/mL
Lyme antibodies	0.43 LI	< 0.90 LI
Treponema pallidum antibody	Nonreactive	nonreactive
Total CK	61 U/L	24–195 U/L
HIV	Negative	Negative
Anti-Neuronal Nuclear Ab, Type 1, 2, 3	Negative	< 1:240 titer
Anti-Glial Nuclear Ab, Type 1	Negative	< 1:240 titer
Purkinje Cell Cytoplasmic Ab Type 1, 2, Tr	Negative	< 1:240 titer
Amphiphysin Antibody	Negative	< 1:240 titer
CRMP-5, IgG	Negative	< 1:240 titer
Striational Antibody	Negative	< 1:120 titer
P/Q-Type Calcium Channel Ab	0.00	<=0.02 nmol/L
N-Type Calcium Channel Ab	0.00	<=0.03 nmol/L
ACh Receptor (Muscle) Binding Ab	0.00	<=0.02 nmol/L
AChr Ganglionic Neuronal Antibody	0.00	<=0.02 nmol/L
Neuronal (V-G) K+ Channel (CASPR2, LG11) Antibody	0.01	<=0.02 nmol/L
Anti GAD-65 Ab	0.00	<=0.02 nmol/L

There was no evidence of volume depletion based on objective bioimpedance measures of total body water and extracellular water. On formal autonomic testing (Finapres NOVA, Finapres Medical Systems, Enschede, Netherlands), he had a low supine resting heart rate (51 bpm) and BP (91/50 mmHg). Slow deep breathing revealed blunted amplitude (4.5 bpm [normal > 7 bpm]) at a low heart rate range (45–52 bpm). Valsalva maneuver resulted in normal heart rate responses (Valsalva ratio 1.38–1.57 [normal > 1.29]) but a “flat top” blood pressure profile and absent phase 4 overshoot. This constellation of findings was indicative of significant sympathetic dysfunction and resultant parasympathetic predominance. His cold-pressor test resulted in only a modest rise in blood pressure (91/52 to 108/63 mmHg [normal: BP increase by > 20/10 mmHg]) and no change in heart rate, also indicative of poor sympathetic reserve. On orthostatic testing, systolic blood pressure dropped from supine average of 92 mmHg to 68 mmHg within 30 s of standing, and further down to 57 mmHg by the 50th second, at which time we terminated the test. His heart rate increased from 49 at baseline to 63 bpm at termination of orthostasis. Peripheral resistance averaged 860 dyn.s.cm− 5 at baseline and increased only minimally (to ~ 990 dyn.s.cm− 5) with standing. Concomitantly, stroke volume decreased from 83 ml supine to 54 ml at the end of standing (50 s), suggesting excessive venous pooling. His hypotension, chronotropic incompetence, suboptimal baroreflex-mediated responses, impaired increase in vascular resistance and significant venous pooling all indicated a loss of sympathetic tone, consistent with acute autonomic dysfunction due to an acute autonomic ganglionopathy which, in his case, was presumed to be autoimmune in nature given its development while on ICI.

**Fig. 1:**
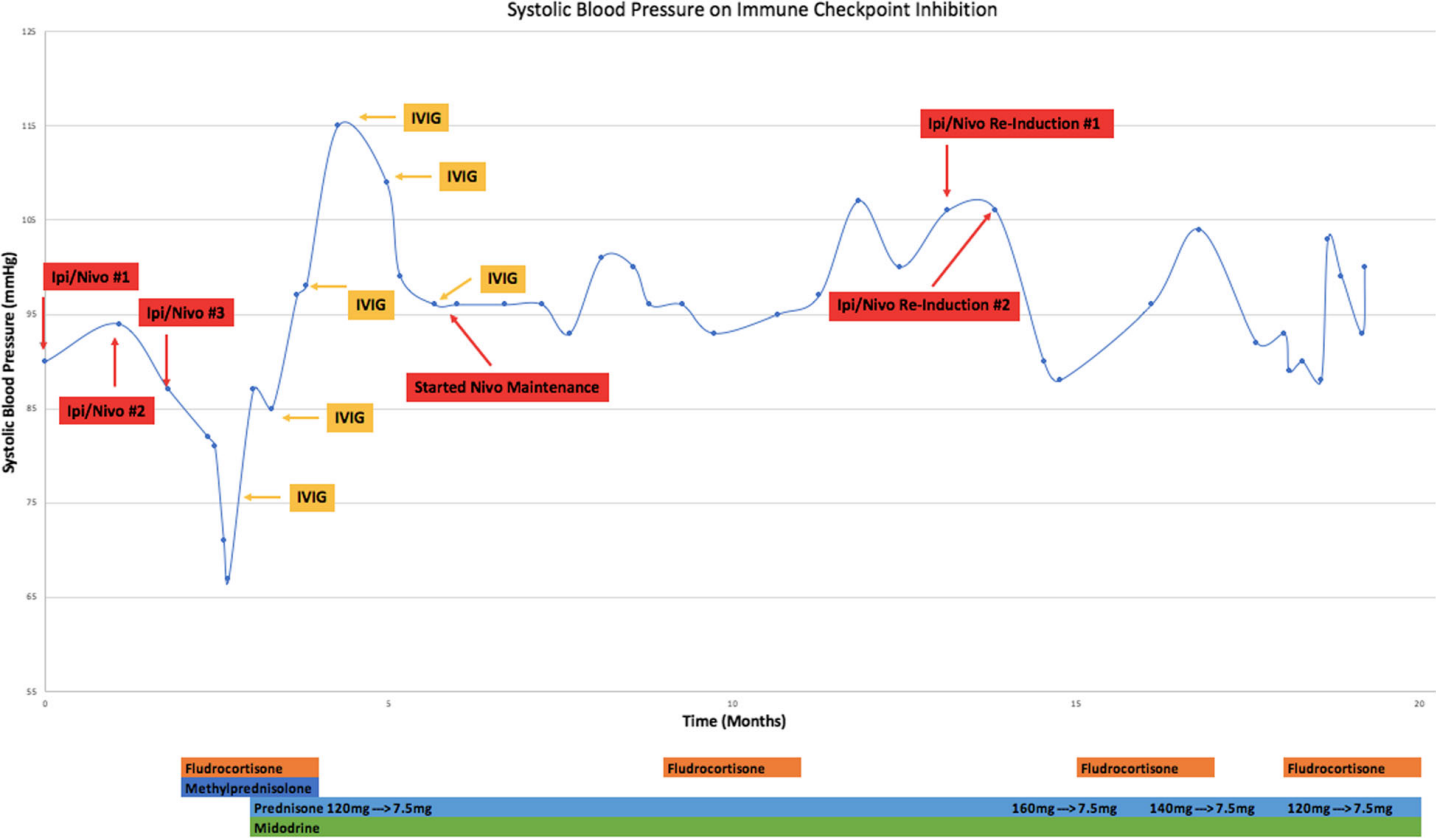
Laboratory testing at first presentation of symptoms

Treatment was initiated with pulse dose solumedrol 1 g IV for 6 days that was converted to oral prednisone upon hospital discharge and was slowly tapered over months. Although there was no serologic evidence of the presence of autoantibodies, seronegative cases of autoimmune autonomic ganglionopathy (AAG) have been shown to respond to intravenous immune globulin (IVIg) [10, 11]. Thus in addition to steroids, IVIG was administered initially as 0.4 g/kg daily for 5 days (total dose 2 g/kg), followed by 1 g/kg every 2 weeks as maintenance (Fig. 1). He was also maintained on midodrine (up to 20 mg three times daily), fludrocortisone (up to 0.3 mg per day in divided doses) and sodium chloride tablets (1 g three times daily). His blood pressure gradually improved to 100/60s mmHg and several months later fludrocortisone and salt were tapered off, midodrine dose was tapered down, IVIg was discontinued, and he was maintained on prednisone 7.5 mg daily, with systolic blood pressures ranging 100–120 s mmHg and minimal orthostatic changes on variable doses of midodrine as the sole treatment agent.

Restaging CT scans approximately 4 months after the last dose of combined ICI showed disease progression in the lung and anal region for which he underwent re-excision of the anal mass followed by palliative radiation. Nivolumab alone was restarted which was well tolerated and he did not have reoccurrence of AAG at this time. Six months later CT scans showed disease progression and he was re-induced with combination ICI, but with only 1 mg/kg of ipilimumab and 3 mg/kg of nivolumab. This led to a milder exacerbation of his orthostatic hypotension, which responded to 2 mg/kg of prednisone followed by a slow taper. After his symptoms improved, he was re-challenged with nivolumab alone but again became hypotensive and ICI was discontinued. He also developed autoimmune transaminitis that was rapidly responsive to oral prednisone. Other treatment options were offered, however he opted for hospice care and ultimately passed away from disease progression.

## Discussion and conclusions

Dual ICI has improved clinical outcomes in advanced melanoma but often requires treatment delays and/or discontinuation due to irAEs [12]. While the most common irAEs of any grade are gastrointestinal (i.e. diarrhea 45%, colitis 13%, hepatitis 20%), skin (i.e. rash 30%, pruritus 35%), and endocrinopathies (hypothyroidism 17%, hypophysitis 7%), neurologic irAEs are far less common (~ 3%), are infrequently reported in ICI clinical trials, and are only more recently being recognized and reported. Polyneuropathies, Guillain-Barré syndrome, transverse myelitis [9], enteric neuropathy manifesting as constipation [13, 14], and myasthenia gravis with or without the presence of acetylcholine receptor antibodies have been described in case reports [15–18]. The rate of neurotoxicity attributable to ICI is rare, only 1–3%, however it is likely an underrecognized complication [19]. While the typical irAEs have a more identifiable symptomatology and time course in relation to ICI, neurotoxicity has variable manifestations making its diagnosis a challenge. Suspicious neurologic symptoms should prompt a comprehensive work-up because exclusion of alternative diagnoses and timely recognition is important to initiate the correct treatment. Patients should be managed in multidisciplinary fashion with input from neurologic specialists. Unfortunately, there are no clinically validated assays that can predict patients who are at risk for neurotoxicity or other irAEs, however this is currently an area of active research.

AAG can have a range of symptoms, many of which are quite debilitating [20] as was the case with our patient. Common symptoms may include sympathetic dysfunction manifesting as orthostatic hypotension, syncope, and anhidrosis, parasympathetic dysfunction such as dry eyes and dry mouth, and/or enteric dysfunction including constipation and gastroparesis [21]. Antibodies associated with AAG include most commonly the ganglionic nicotinic acetylcholine receptor antibodies [22], and cases have been described in paraneoplastic syndromes (several antibodies described, most commonly anti-neuronal antibody type 1 antibodies, also known as anti-Hu antibodies). However seronegative cases have also been reported [22] and it is thought that other auto-antibodies, potentially not yet discovered, are contributory in such cases [21, 23]. While the serologic testing in this patient was negative, the AAG was still attributed to an autoimmune mechanism given the clinical setting and in the absence of other apparent causes other than the melanoma itself and the ICI treatment.

AAG in non-ICI-induced settings is treated with steroids, IVIG, or plasma exchange, and there is some evidence that mycophenolate and rituximab may be used in patients who are refractory of these initial immunomodulatory agents [24]. This overlaps with the general principle of immunomodulatory agents recommended for treatment of ICI-induced irAEs and was successful in treating our patient [25, 26]. Case series have demonstrated that IVIg can be efficacious for AAG, as it was here, irrespective of whether antibodies against the acetylcholine receptor are detected or not [10, 11]. For example, of 6 AAG patients treated with immunosuppressive therapy in a case series reported by Iodice et al., patients with both seropositive (*n* = 3) and seronegative (*n* = 2) AAG responded to IVIg. Conversely, we acknowledge that in another case series, 4 seronegative AAG patients treated with IVIg achieved little to no clinical benefit, while high dose intravenous steroids seemed to induce the best responses in those cases [27]. Despite the limited data on treatment of AAG, IVIg is considered a first line therapy since it is an antibody-mediated process and was the reason it was selected as a treatment in this case, in addition to high dose intravenous steroids.

Careful thought has to be given to re-inducing patients with ICI who have had prior irAEs. In this case, our patient made a significant, albeit partial recovery of his AAG and the risk of his melanoma progression was felt to outweigh the risk of developing additional toxicity. Fortunately, when his dysautonmia recurred with reinduction of dual ICI, the symptoms were milder and were managed on an outpatient basis with a prednisone taper in addition to the adjunct use of midodrine and fludrocortisone.

Hypothetically, the autoimmunity causing AAG and other ICI-related toxicities is triggered by autoreactive T-cells. Blocking PD-1 and CTL4 can disrupt important self-tolerance balances, thereby causing a host of autoimmune pathologies. It has also been theorized that autoantibodies could be formed as a result of T-cell dependent activation of B-cells. For example, a case of ICI-associated Guillain-Barre syndrome describes a pan-dysautonomia that occurred after a single dose of ipilimumab – the patient had a tonically dilated pupil, gastrointestinal dysmotility, urinary retention, and orthostatic hypotension. Electromyography showed Guillan-Barre-like changes. The patient recovered with IVIG and droxidopa [28]. Other hypotheses for neurotoxicity from ICI have included inflammation of endoneurial microvessls and sub-perineural inflammation and edema [29]. In the case of AAG, auto-antibodies can be detected but are not mandatory for diagnosis in the correct clinical setting.

In summary, we report a case of AAG induced by dual ICI therapy, requiring high dose solumedrol and IVIG, which recurred with re-induction of ICI. While this is a rare neurotoxicity related to ICI use, clinicians should maintain high suspicion for this toxicity in a patient on ICI who presents with refractory hypotension, nausea, and other dysautonomic symptoms.

## Data Availability

Not applicable.

## Abbreviations

AAG: Autoimmune autonomic ganglionopathy
CTLA-4: Cytotoxic T-lymphocyte-associated antigen-4
ICI: Immune checkpoint inhibitors
irAE: Immune-related adverse events
IVIG: Intravenous immune globulin
PD-1: Programmed-death-1
